# Stereotactic lung reirradiation for local relapse: A case series

**DOI:** 10.1016/j.ctro.2021.03.007

**Published:** 2021-04-05

**Authors:** Rémy Kinj, Alessio Casutt, Alexander Bennassi, Hasna Bouchaab, Véronique Vallet, Alban Lovis, Mahmut Ozsahin

**Affiliations:** a Department of Radiation Oncology, Lausanne University Hospital and University of Lausanne, Lausanne, Switzerland; b Department of Pulmonology, Lausanne University Hospital, Lausanne, Switzerland; c Department of Medical Oncology, Lausanne University Hospital and University of Lausanne, Lausanne, Switzerland; d Institute of Radiation Physics, Lausanne University Hospital and University of Lausanne, Lausanne, Switzerland

**Keywords:** Lung reirradiation, SBRT reirradiation, Stereotactic treatment, Lung cancer

## Abstract

**Introduction:**

Local recurrence after lung SBRT for early stage NSCLC is rare but its treatment remains a challenge due to limited surgical options. We report a case series of 5 patients treated by stereotactic lung salvage reirradiation for local relapse after a previous lung SBRT.

**Material and methods:**

Included patients presented an isolated primary lung relapse within at least the 50% isodose of the previous SBRT treatment. Typical reirradiation schedule was 60 Gy in 8 fractions at isodose 80% and was delivered by Cyberknife® using Synchrony® fiducial tracking system. Dose summations were performed to evaluate the safety of the reirradiation.

**Results:**

We identified 5 patients presenting peripheral lesions. All reirradiated lesions were locally controlled after a median follow-up of 11.1 months (6,7–12,2), while PFS at 6 months was 60% (n = 3). We did not notice any Grade 3 or more acute or late adverse event.

**Conclusion:**

We observed encouraging short-term outcome of lung SBRT reirradiation in patients presenting isolated local relapse of an early-stage NSCLC. Further studies are necessary to confirm the safety and efficiency of this salvage treatment approach.

## Highlights

• Stereotactic body radiotherapy is a well-recognized option for treatment of early stage non-small cell lung cancer.
• Locale relapse is rare but still remains a challenge to manage.
• SBRT lung reirradiation is uncommon and few studies report its technique and the outcome.
• SBRT lung reirradiation may be considered in an appropriate setting and in a well selected population.

## Introduction

Stereotactic body radiotherapy (SBRT) is a well-established treatment option for patients presenting an early-stage non-small cell lung cancer (NSCLC). The main failure pattern after lung SBRT is represented by distant failure. Local recurrence in a previously irradiated lung volume is observed in 5 to 15% of cases and treatment remains a challenge due to co-morbidities limiting surgical options [1]. Retreatment with conventional fractionated radiotherapy (CFRT) can be consider as a salvage option but remains poor outcomes [2]. Few studies explored salvage SBRT for reirradiation after a first course of lung SBRT, more experiences must be described to determine control and toxicity rates. We recently considered the opportunity to repeat lung SBRT. Here we report a case series of 5 patients treated by stereotactic lung salvage reirradiation for local relapse after a previous lung SBRT.

## Material and methods

From November 2019, during our clinical follow-up, we identified patients who presented localized lung relapses in a previously irradiated volume by SBRT. We included in this report patients presenting an isolated primary lung relapse within at least the 50% isodose of the previous SBRT treatment. The decision to reirradiate was approved by the local multidisciplinary thoracic tumor board, and patients were not considered eligible for salvage surgery due to co-morbidities. All patients benefited from reirradiation after fiducial marker (FM) placement and was performed by Cyberknife® using Synchrony® fiducial tracking system (Accuray, Sunnyvale). Typical reirradiation schedule was 60 Gy in 8 fractions at isodose 80% corresponding to a Biological Effective Dose for α/β 10 Gy (BED10) of 105 Gy. We performed deformable registration on Raystation® (Raysearch, Stockholm) treatment planning system, then plans were summed in order to evaluate the safety of the reirradiation treatment. Cumulative dosimetry had to respect the constraints of a 5 fractions plan for organs at risk according to American Association of Physicists in Medicine doses constraints [3]. Cumulative dosimetry had particularly to respect firstly spinal canal constraints (V23 < 0.03 cc), secondly and lungs tissues constraints V5 < 65%,and finally both V12.5 < 1500 cc,V13.5 < 1000 cc. There was no maximal dose limit in lungs while maximal dose had to be localized in the PTV of the reirradiated target.

## Results

We identified 5 patients, 3 out of 5 relapses were histologically proven, and the remaining two were assessed by their clinical evolution followed by iterative morphological and metabolic imaging.

The median age of patients at relapse was 78.9 years (range, 62.6–88.8) and relapse was diagnosed with a median time lapse of 31.3 months (range, 15.4–91.6) after the first SBRT. First SBRT median dose was 55 Gy (45–60) in 5 fractions [3], [4], [5], [6], [7], [8]. Most of first lung cancers (4/5) were classified as cT1N0M0 (8th TNM classification). The remaining last patient was firstly irradiated for an isolated lung relapse of a lung adenocarcinoma previously (20 months before the radiation therapy) treated by surgical resection.

The mean lung dose (MLD) of the reirradiation was 1.9 Gy (range, 1.0–2.0), the volume of lungs receiving 5 Gy (V5) was 7.5% (range, 4.9–9.5) and V20 1% (range, 0.8–3.9). All reirradiated lesions were peripherally located and the median Planning Target Volume (PTV) was 4.0 mL.

Three patients previously received Cyberknife® treatment with fiducial tracking, one patient had Tomotherapy® SBRT, and one had a VMAT SBRT treatment.

After reirradiation, cumulative MLD was 5.1 Gy (range, 3.6–7.8) and cumulative V5 was 28.5 Gy (range, 16.0–48.5). Median maximal cumulated PTV dose was 90.8 Gy (range, 76.2–135.8) (Table 1).

**Table 1:** Patients and treatment characteristics.

Patients Number	Age (years)	Performans Status	Histology	Time to relapse (Months)	First course regimen (Gy/number of fractions)	Second course regimen (Gy/number of fractions)	PTV Maximal physical dose summation (Gy)	Mean Lung Dose (Gy)	Cumulative mean lung dose (Gy)	Volume of Lung receiving 5 Gy (%)	Cumulative Volume of Lung receiving 5 Gy (%)
N-1	82,6	1	SCC	93,4	60/8	60/8	135,3	1,6	7,3	7,5	32,0
N-2	88,8	2	SCC	31,3	55/5	50/5	90,3	1,9	5,1	7,3	28,5
N-3	75,2	1	ADK	37,5	54/3	60/8	90,8	1,1	4,1	4,9	16,0
N-4	78,9	2	SCC	15,6	45/3	35/5	76,2	2,0	7,8	9,0	48,5
N-5	62,6	1	ADK	21,4	55/5	60/8	101,1	2,0	3,6	9,5	19,0

SCC: Squamous cell cancer.

ADK: Adenocarcinoma.

All reirradiated lesions were locally controlled after a median follow-up of 11.1 months (6,7–12,2), while PFS at 6 months was 60% (n = 3). One patient presented at 6 months a contralateral new lung lesion successively treated by SBRT. Another patient developed a single brain metastasis that was surged and irradiated. We did not notice any Grade 3 or more acute or late adverse event (Common Terminology Criteria for Adverse Events version 4) [4]. Most frequent adverse event was acute Grade 1 asthenia in 2 patients.

## Discussion

We report our early clinical outcome after lung SBRT reirradiation. It may represents a new salvage option for these non-operable patients with significant co-morbidities.

The singularity of our data is that we report a series of patients who underwent two sequences of lung high-dose SBRT closely located. Most of publications concerning SBRT reirradiation for local relapse usually report data after a single or two conventionally-fractionated course/s. These experiences revealed acceptable local control rate with relatively high rates of lung toxicity such as radiation induced pneumonitis [5], [6], [7], [8], [9]. Synchrony® tracking system uses a 3-D co-ordinate system that tracks the target during the respiratory cycle by means of previously inserted metallic FM. The tracking system permits the maximal reduction of margins conducing to a better sparing of healthy lung tissue. As reirradiation volume always correlates with toxicity outcome in the reirradiation setting, our accurate SBRT technique permits a better toxicity outcome. Moreover we choosed a fractionated regimen in 8 fractions (usually used for ultra-central lesions) in order to enhance tolerance of treatment while persevering biological efficiency ((BED10) of 105 Gy).

Kennedy *et al*. also used accurate SBRT technique in a comparable study population. Their experience represents the largest cohort with 21 included patients. They remained a low rate of Grade 2 lung toxicity (10% of pneumonitis) and no Grade 3 toxicity while local control was 81% at 2 years.

John *et al,* recently published a retrospective multicenter (n = 8) study focused on patients (n = 27) treated by two courses of lung SBRT. The reported one-year local control rate was 78.3% and no grade > 2 toxicity was observed. However, the reported delivered doses were inferior to those of our series. In particular, the first course median dose was 38.5 Gy in 5 fractions while the median reported dose was 40 Gy in 5 fractions [10].

**Fig. 1:**
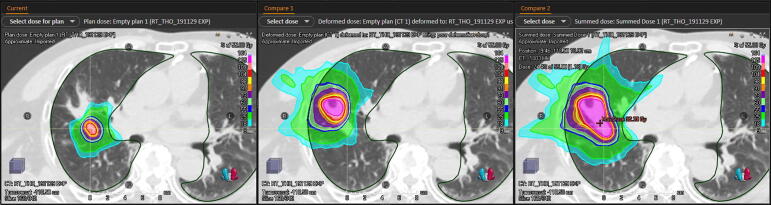
Dosimetric presentation of patient N-2. Left: current relapse treatment of 50 Gy in 5 fractions. Middle: previous SBRT irradiation dose of 55 Gy in 5 fractions registered using deformable registration on current CT. Right: cumulative dosimetry of treatments with maximal point dose of 92.4 Gy.

Hear *et al* reported a series of 10 patients that benefited of SBRT reirradiation with BED10 doses ≥ 100 Gy, this treatment was considered as a viable salvage option for inoperable locally recurrent NSCLC [11]. Nishimura *et al* also reported the cases of two elderly patients successfully treated by salvage lung SBRT reirradiation [12]. In our experience, we performed cumulative physical dose summation to evaluate the safety of the reirradiation. Cumulative dose had to respect the most restrictive constraints template for stereotactic radiotherapy (Fig. 1). As an example, if the first course was delivered in 5 fractions and the reirradiation was delivered in 8 fractions, the composite plan had to respect the constraints of a 5 fraction plan for organs at risk according to American Association of Physicists in Medicine doses constraints [3]. Moreover, we collected data concerning MLD and V5 and tried to optimize their values, as higher values seem to be part of complications occurrence, and we did not observed any Grade 3 or more toxicity at last news [13], [14]. We can notice that we did not treat central or ultra-central tumors directly invading bronchial tree [15]. The main limitation of the report is the low number of patients, however our results seems to correspond to the previously published data.

## Conclusion

In our preliminary experience, we observed short-term favorable outcome of lung SBRT reirradiation in patients presenting isolated local relapse of an early-stage NSCLC. Further studies are necessary in order to establish if this approach could be considered a safe and effective salvage treatment.

## Declaration of Competing Interest

The authors declare that they have no known competing financial interests or personal relationships that could have appeared to influence the work reported in this paper.
